# Vertigo

**Published:** 1890-03-01

**Authors:** 


### VERTIGO.

Dr. Buzzard's recent address to the Harveian Society of London, on "Vertigo of Bulbar Origin," demands notice. Vertigo is a symptom which may occur in connexion with many conditions. Paroxysms of vertigo occurring in a person of previously fair health, and accompanied by deafness or noises, are now constantly attributed to disease of the labyrinth of the ear. But Dr. Buzzard's impression is that it is through its centre in the medulla oblongata that the auditory nerve is affected, and not at its periphery. Some irritative influence in the bulb may be the determining agent in the production of giddiness and other troubles, possibly uric acid salts. Dr. Buzzard has long prescribed salicylate of sodium in cases of vertigo associated with auditory nerve symptoms, on account of its influence in the elimination of uric acid, and also because it apparently exerts some direct influence upon centres in the medulla oblongata.

